# Gut microbiota regulates circadian oscillation in hepatic ischemia–reperfusion injury-induced cognitive impairment by interfering with hippocampal lipid metabolism in mice

**DOI:** 10.1007/s12072-023-10509-w

**Published:** 2023-04-01

**Authors:** Zhigang He, Yanbo Liu, Zhen Li, Tianning Sun, Zhixiao Li, Anne Manyande, Hongbing Xiang, Jun Xiong

**Affiliations:** 1 Department of Anesthesiology and Pain Medicine, Tongji Hospital, Tongji Medical College, Huazhong University of Science and Technology, Wuhan, China; 2 Department of Emergency Medicine, Tongji Hospital, Tongji Medical College, Huazhong University of Science and Technology, Wuhan, China; 3 Department of Critical Care Medicine, Tongji Hospital, Tongji Medical College, Huazhong University of Science and Technology, Wuhan, China; 4 School of Human and Social Sciences, University of West London, London, UK; 5 Hepatobiliary Surgery Center, Union Hospital, Tongji Medical College, Huazhong University of Science and Technology, Wuhan, China

**Keywords:** Gut microbiota, Circadian oscillation, Hepatic ischemia–reperfusion injury, Cognitive disorders, Dysbiosis, Fecal microbiota transplantation, Hippocampus, Fecal metabolites, Lipid metabolism, Metabolomics

## Abstract

**Background:**

Hepatic ischemia–reperfusion injury (HIRI) is a common complication of liver surgery, which can lead to extrahepatic metabolic disorders, such as cognitive impairment. Recent observations have emphasized the critical effects of gut microbial metabolites in regulating the development of liver injury. Herein, we investigated the potential contribution of gut microbiota to HIRI-related cognitive impairment.

**Methods:**

HIRI murine models were established by ischemia–reperfusion surgery in the morning (ZT0, 08:00) and evening (ZT12, 20:00), respectively. Antibiotic-induced pseudo-germ-free mice were gavaged with fecal bacteria of the HIRI models. Behavioral test was used to assess cognitive function. 16S rRNA gene sequencing and metabolomics were used for microbial and hippocampal analysis.

**Results:**

Our results established that cognitive impairment caused by HIRI underwent diurnal oscillations; HIRI mice performed poorly on the Y-maze test and the novel object preference test when surgery occurred in the evening compared with the morning. In addition, fecal microbiota transplantation (FMT) from the ZT12-HIRI was demonstrated to induce cognitive impairment behavior. The specific composition and metabolites of gut microbiota were analyzed between the ZT0-HIRI and ZT12-HIRI, and bioinformatic analysis showed that the differential fecal metabolites were significantly enriched in lipid metabolism pathways. After FMT, the hippocampal lipid metabolome between the P-ZT0-HIRI and P-ZT12-HIRI groups was analyzed to reveal a series of lipid molecules with significant differences.

**Conclusions:**

Our findings indicate that gut microbiota are involved in circadian differences of HIRI-related cognitive impairment by affecting hippocampal lipid metabolism.

**Graphical abstract:**

**Supplementary Information:**

The online version contains supplementary material available at 10.1007/s12072-023-10509-w.

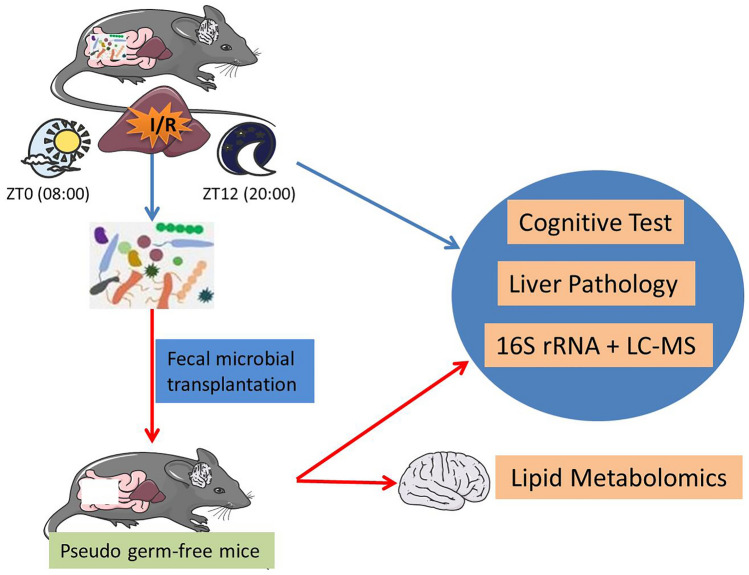

## Introduction

Hepatic ischemia–reperfusion injury (HIRI) is one of the most common complications in the perioperative period, especially in liver surgery and transplantation [1]. In addition to influencing the hospitalization period and perioperative mortality, HIRI may also impair cognitive function in some patients, causing problems to their long-term quality of life [2]. Previous animal model studies have shown that liver injury may interfere with ammonia metabolism, causing hyperammonemia and hepatic encephalopathy, which affect cognitive function by inducing central inflammation and affecting central metabolism [3]. However, the specific mechanism of cognitive impairment caused by HIRI remains unclear.

In recent years, more and more attention has been paid to the role of gut microbiome in cognitive dysfunction. Gut microbiota plays an important role in the pathophysiological processes of various cognitive impairments related to obesity, diabetes, the postoperative phase, post-stroke, and Alzheimer’s disease [4]. Gut microbiota can influence host cognitive function impairment by regulating blood–brain barrier permeability, brain energy homeostasis, and synaptic transmission through the brain–gut axis [5]. Numerous studies have revealed associations between the gut microbiome and various types of acute liver injury (ALI) [6], induced by drugs, d-galactosamine, alcohol, and viral hepatitis. In addition, antibiotic pretreatment has been shown to alleviate HIRI and orthotopic liver transplantation outcomes in mice and humans [7], and the gut microbial metabolite to participate in the pathophysiological process of HIRI [8].

Cognitive impairment is strongly associated with circadian rhythms in both humans and animal models [9]. Liver activities, such as digestion and absorption, synthesis and uptake, assimilation, and detoxification, show significant diurnal changes, which enable their alignment with energy metabolism [10]. It is also well documented that gut microbiota undergoes diurnal oscillations both at the compositional and functional levels [11]. But while gut microbial metabolite, 1-phenyl-1,2-propanedione is involved in regulating the diurnal variation of hepatotoxicity induced by acetaminophen [12], the gut microbial metabolite, 3,4-dihydroxyphenylpropionic acid is involved in regulating the diurnal variation of HIRI [8]. However, whether the gut microbiome is implicated in cognitive impairment associated with HIRI remains unknown.

In the present study, we explored the mechanism of the diurnal variation of HIRI-related cognitive impairment in mice, by focusing on the gut microbiota.

## Materials and methods

### Animals

Male-specific pathogen-free (SPF) C57BL/6 mice, aged 6–8 weeks, were housed in a temperature-controlled colony room under the standard 12 h light/dark conditions (8:00, light on; 20:00, light off), with free access to food and water ad libitum. Hepatic ischemia–reperfusion injury surgery was performed at ZT0 (8:00) or ZT12 (20:00). All mice were obtained from the Beijing Vital River Laboratory Animal Technology Co., Ltd., (Beijing, China), and all experimental procedures commenced following the approval of the Institutional Animal Care and Use Committee in Tongji Hospital, Huazhong University of Science and Technology.

### The mouse model of hepatic ischemia–reperfusion injury

Hepatic ischemia–reperfusion injury surgery was performed at ZT0 or ZT12 (ZT0-HIRI, *n* = 6; ZT12-HIRI, *n* = 6; ZT0-Ctr, *n* = 6; ZT12-Ctr, *n* = 6). After anesthesia with 1% pentobarbital sodium (50 mg/kg, intraperitoneal), mice were placed on an operating table, the abdominal skin was disinfected, and a median incision made to expose the liver area. The hepatic ischemia–reperfusion surgery was performed according to the method previously described [8]. Briefly, ischemia was induced by occluding the left hepatic artery and portal vein for 90 min using an artery clamp. No vascular occlusion is in sham-controlled mice. During the operation, the mice were kept warm, and the surgical incision is treated with analgesia after the operation. Behavioral tests were performed 72 h after reperfusion, and feces, liver tissue, serum, and hippocampal tissue were collected for subsequent experiments and detection.

### Behavioral test

For the HIRI and control groups, behavioral experiments were performed 72 h after reperfusion. For the pseudo-germ-free mice group, behavioral experiments were performed during the next day after completion of the microbiota transfer. The open-field test, the Y-maze test, and the novel object preference test were performed to assess cognitive function. The test apparatus was wiped with 75% ethanol to eliminate odor after each experiment.

### Open field test

As previously described [13], the mice were placed into the center of a black open-field chamber (L × W × H: 40 cm × 40 cm × 40 cm) after habituation. They moved freely under dim light (300 lx) for 5 min, and the total distance traveled and the time spent in the center area were analyzed.

### Novel object preference test

Two identical objects were placed at two corners 6 cm from each border as previously described [13]. In the training stage, the animal was allowed to explore freely for 5 min after accommodation without objects, and the total exploration time around each object was recorded. After 2 h, the mice were placed again in the same apparatus and allowed to freely explore for 5 min with one of the identical objects being replaced with a novel object (test session). The exploration time around the novel (NT) and familiar objects (FT) was recorded and used to calculate the recognition index.

### Y-maze test

As previously described [13], the Y-maze was performed with two stages in a Y-shaped compartment with three identical arms, each at an angle of 120° (L × W × H: 30 cm × 8 cm × 15 cm). The start arm (animal entry) and the common arm were always kept open, and the new arm was blocked in the first stage. Then, the mice explored the two open arms for 5 min. After 2 h, all the arms were opened, and the mice had free access to the three arms for 5 min (test trial). We recorded the time spent within each arm, the number of mice entering the new arm, and the total distance traveled by the mice for analysis.

### Hematoxylin and eosin (HE) staining

Liver specimens were processed using standard HE procedures, including steps, such as dehydration, embedding, sectioning, and tissue staining. The staining was scanned on each slide and the degree of liver damage was graded using the Suzuki’s score [8].

### Pseudo-germ-free mice and fecal microbial transplantation

Microbiota depletion and fecal microbiota transplantation (FMT) were performed as described previously [8]. For pseudo-germ-free mice, C57BL/6 mice received vancomycin (100 mg/kg), neomycin sulfate (200 mg/kg), metronidazole (200 mg/kg), and ampicillin (200 mg/kg) intra-gastrically once daily for 4 days. For fecal microbial transplantation, donor mice feces from the ZT0-Ctr, ZT0-HIRI, ZT12-Ctr, and ZT12-HIRI groups were collected and re-suspended in phosphate-buffered saline (PBS) to a concentration of 0.125 g/mL. This suspension was administered to mice via oral gavage (0.15 mL) daily for 3 days. Pseudo-germ-free mice receiving FMT were divided into four groups: P-ZT0-Ctr (*n* = 6), P-ZT0-HIRI (*n* = 6), P-ZT12-Ctr (*n* = 6), and P-ZT12-HIRI (*n* = 6), and the behavioral experiments were performed during the next day after FMT.

### 16S rRNA microbiome sequencing

After the behavioral tests, fecal samples were obtained, snap-frozen, and stored at − 80 °C. The 16S rRNA sequencing of the microbiota was performed at the OE biotech Co., Ltd. (Shanghai, China). Fecal samples were subjected to DNA extraction, amplification, library construction, and sequencing (Illumina Inc., San Diego, CA; OE Biotech Company; Shanghai, China) to obtain raw data in FASTQ format. The software and platform (https://cloud.oebiotech.cn/task/) provided by the company were used for further bioinformatic analysis of the raw data.

### LC–MS analysis of fecal metabolites and lipid metabolomics in the hippocampus

The key steps of LC–MS analysis included fecal samples or hippocampus pretreatment, metabolite extraction, full-scan LC–MS detection, data pretreatment, and statistical analysis. The analytical instrument for this experiment was a Dionex U3000 UHPLC LC–MS system consisting of a QE plus high-resolution mass spectrometer, and the original LC–MS data were processed by software Progenesis QI V2.3 (Nonlinear, Dynamics, Newcastle, the UK). Variable Importance of Projection (VIP) values obtained from the OPLS-DA model were used to rank the overall contribution of each variable to group discrimination. A two-tailed Student’s *T* test was further used to verify whether the differences in metabolites between groups were significant. Differential metabolites were selected with VIP values greater than 1.0 and *p* values less than 0.05.

### Statistical analysis

All the quantification data were expressed as means ± SEM, with error bars representing SEM. The normality distribution assessment was evaluated by the Kolmogorov–Smirnov test, and differences among the groups were assessed by one-way analysis of variance (ANOVA), followed by Tukey’s multiple comparisons test, and *p* < 0.05 was considered statistically significant. Statistical analyses and graphs (except for some data from 16S rRNA microbiome sequencing analyses and LC–MS) were performed with the GraphPad Prism 6.0 and Adobe Photoshop 22.1.1.

## Results

### Cognitive impairment caused by HIRI underwent diurnal oscillations

**Fig. 1:**
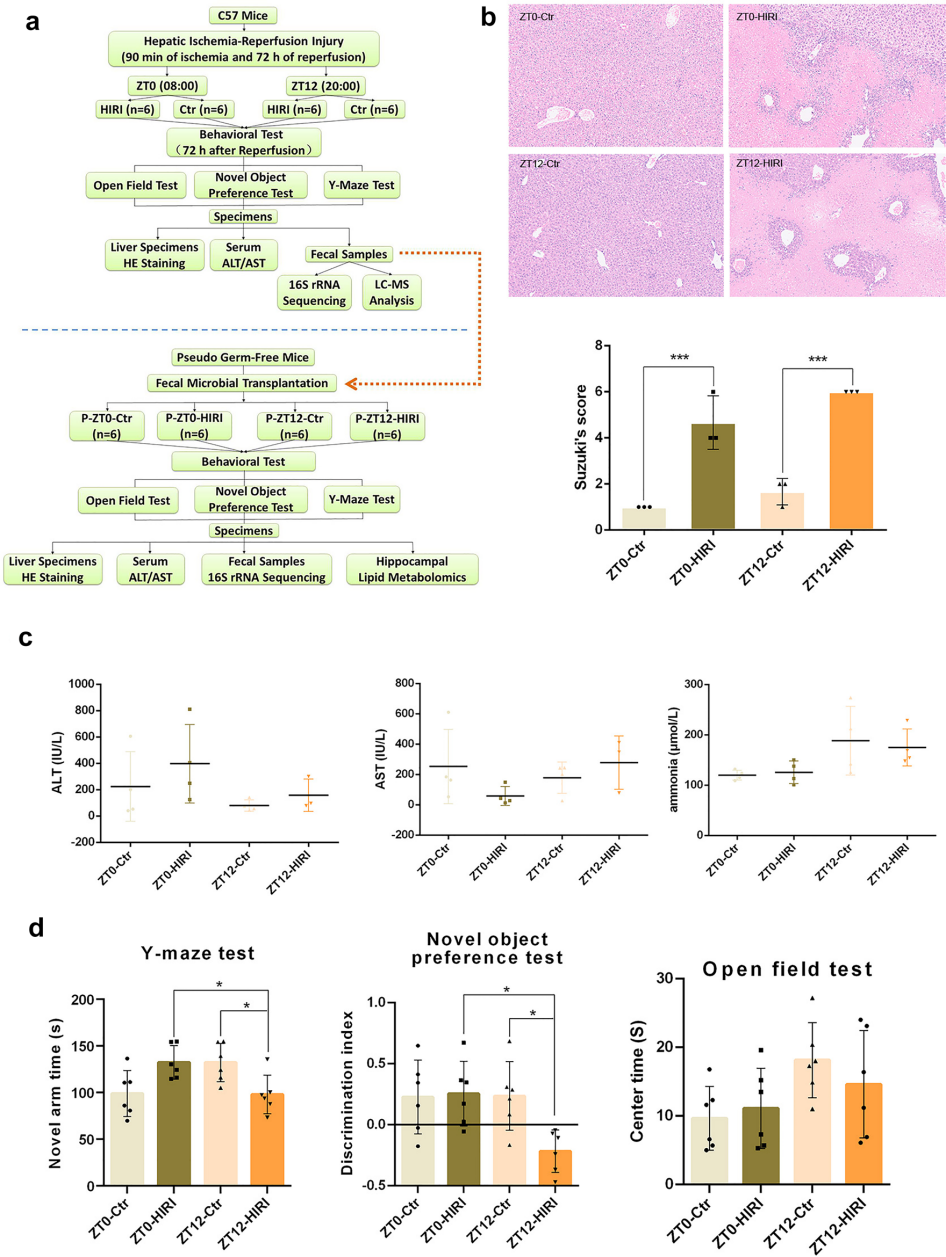
Liver injury and cognitive impairment in the mouse model of hepatic ischemia–reperfusion injury under ZT0/ZT12. Four groups: ZT0-HIRI, *n* = 6; ZT12-HIRI, *n* = 6; ZT0-Ctr, *n* = 6; ZT12-Ctr, *n* = 6. **A** Schematic illustration of the experimental design. **B** The light microscopy and Suzuki’s score of liver tissues (*n* = 3). Hematoxylin and eosin (H&E). Magnification × 100. **C** Serum levels of ALT and AST (*n* = 3–4); Blood ammonia concentration (*n* = 4). **D** Cognitive behavior test (open-field test, Y-maze test and novel object preference test; *n* = 6). One-Way ANOVA (Tukey’s multiple comparisons test); *p < 0.05, ***p < 0.001

Compared with the control mice, the HIRI model showed significant differences in liver pathology (Fig. 1B), but not in serum ALT/AST and ammonia concentration (Fig. 1C). There was no diurnal difference in the liver pathological score, serum ALT/AST, and ammonia concentration (Fig. 1B, C). Compared with the ZT0, the HIRI model mice in the ZT12 group performed poorly on the Y-maze test and the novel object preference test, but not the open-field test (Fig. 1D). HIRI models also performed poorly on the Y-maze test and the novel object preference test compared with the control group when established at ZT12 (Fig. 1D). According to these findings, HIRI surgery caused severe liver injury, and induced more significant learning and short-term memory problems when surgery was performed at ZT12.

### ZT12-HIRI mice showed abnormal gut microbiota composition

**Fig. 2:**
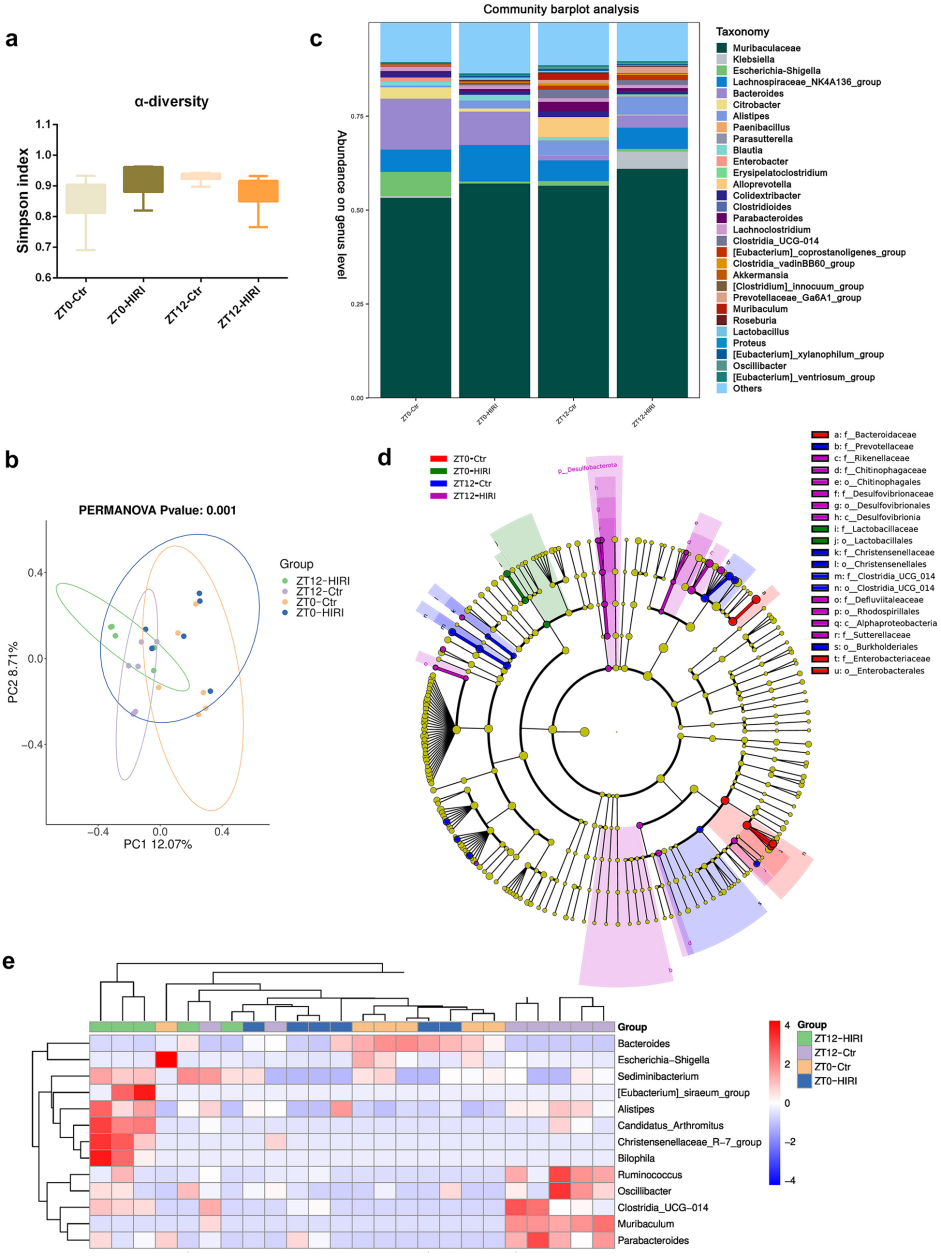
The composition of gut microbiota was different between the ZT0 and ZT12 groups. **A** Alpha Diversity (Simpson index). **B** Beta Diversity (PCoA). **C** The relative abundance of bacteria at the genus level. All genera with an average relative abundance below 1% were grouped to “others.” **D** LEfSe analysis. **E** Heatmap of differential species at the genus level

16S rRNA gene sequencing technology was used to detect the composition of gut microbiota in HIRI mice. For Alpha diversity, the Simpson index did not differ between the four groups (Fig. 2A). For Beta diversity, the principal component analysis (PCA) depicted significant compositional discriminations inside the gut microbiota between the HIRI and control or ZT0 and ZT12, which revealed that gut microbial composition underwent diurnal differences and HIRI altered the microbiota composition (Fig. 2B). In addition, the microbiome composition was considerably altered after HIRI and showed diurnal differences (Fig. 2C, D). Statistical analysis was performed at the genus level, and Heatmap was drawn according to the relative abundance of different species (Fig. 2E).

### Diurnal differences in metabolites of gut microbiota in HIRI mice

**Fig. 3:**
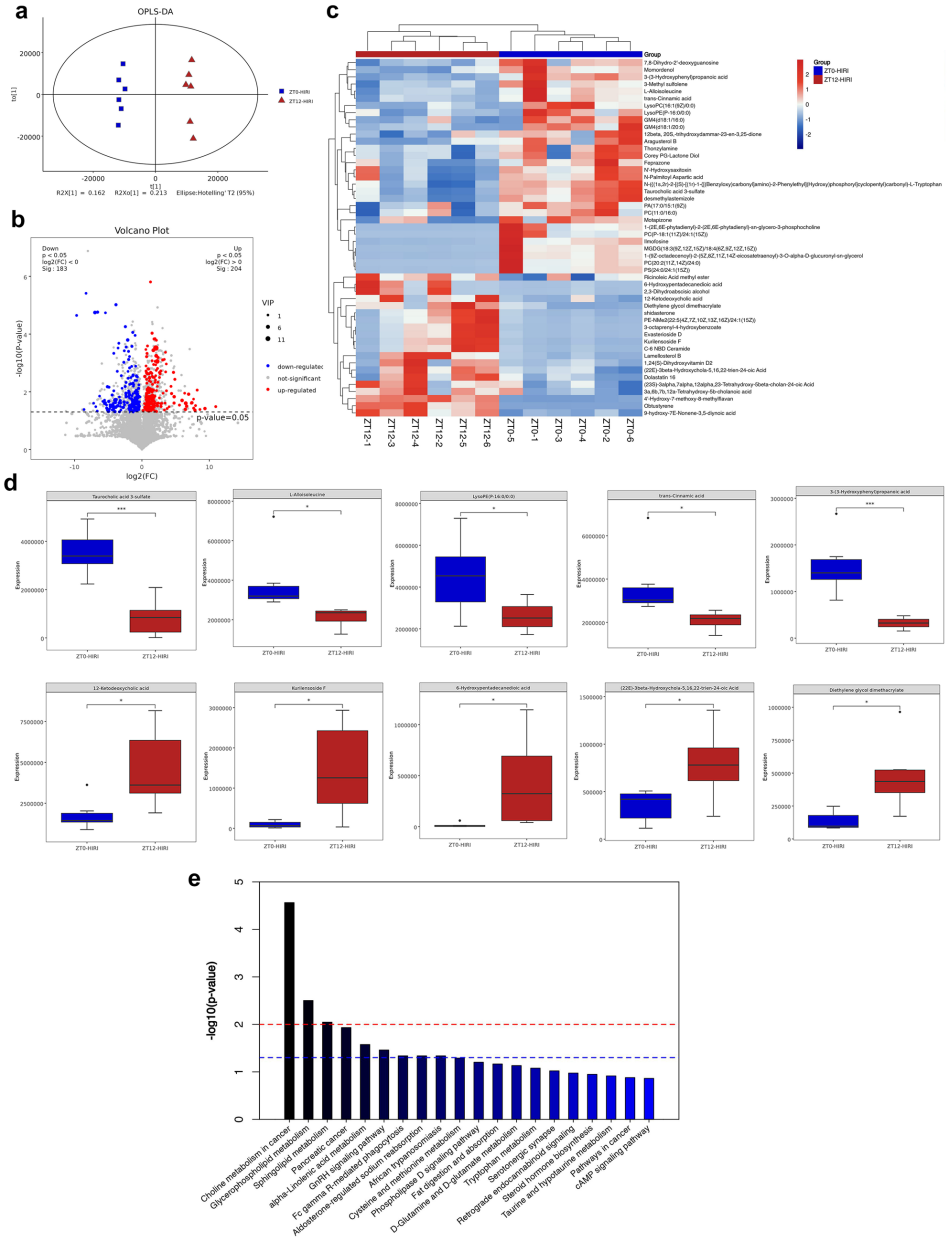
Diurnal differences in fecal metabolites in HIRI mice. **A** OPLS-DA plot. **B** Volcano plot of differential metabolites. **C** Cluster plot of the top 50 differential metabolites. **D** Box plot of the top different metabolites. **E** KEGG pathway enrichment analysis of differential metabolites. T test; *p < 0.05, ***p < 0.001

Liquid chromatography–mass spectrometry (LC–MS) was used to analyze the differences in fecal metabolites between the ZT0-HIRI and ZT12-HIRI groups. There were significant differences in OPLS-DA scores between the two groups (Fig. 3A). Compared with the ZT0-HIRI, a total of 387 metabolites of intestinal flora with significant differences were detected in the ZT12-HIRI group, including 183 which were up-regulated and 204 down-regulated (Fig. 3B). We ranked the differential metabolites based on the Variable Important in the Projection (VIP) and performed a cluster analysis of the top 50 (Fig. 3C). The top 5 down-regulated metabolites of the ZT12-HIRI group according to the VIP were Taurocholic acid 3-sulfate, L-Alloisoleucine, LysoPE(P-16:0/0:0), trans-Cinnamic acid, and 3-(3-Hydroxyphenyl)propanoic acid, while the top 5 up-regulated metabolites were 12-Ketodeoxycholic acid, Kurilensoside F, 6-Hydroxypentadecanedioic acid, (22E)-3beta-Hydroxychola-5,16,22-trien-24-oic Acid, and Diethylene glycol dimethacrylate, respectively (Fig. 3D). In addition, the KEGG pathway enrichment analysis was performed on differential metabolites which were found to be significantly enriched in lipid metabolism pathways, such as the glycero-phospholipid metabolism, sphingolipid metabolism, alpha-linolenic acid metabolism, and fat digestion and absorption (Fig. 3E).

### Fecal microbiota transplantation (FMT) induced cognitive impairment behaviors and reshaped the gut microbiome in pseudo germ-free mice

**Fig. 4:**
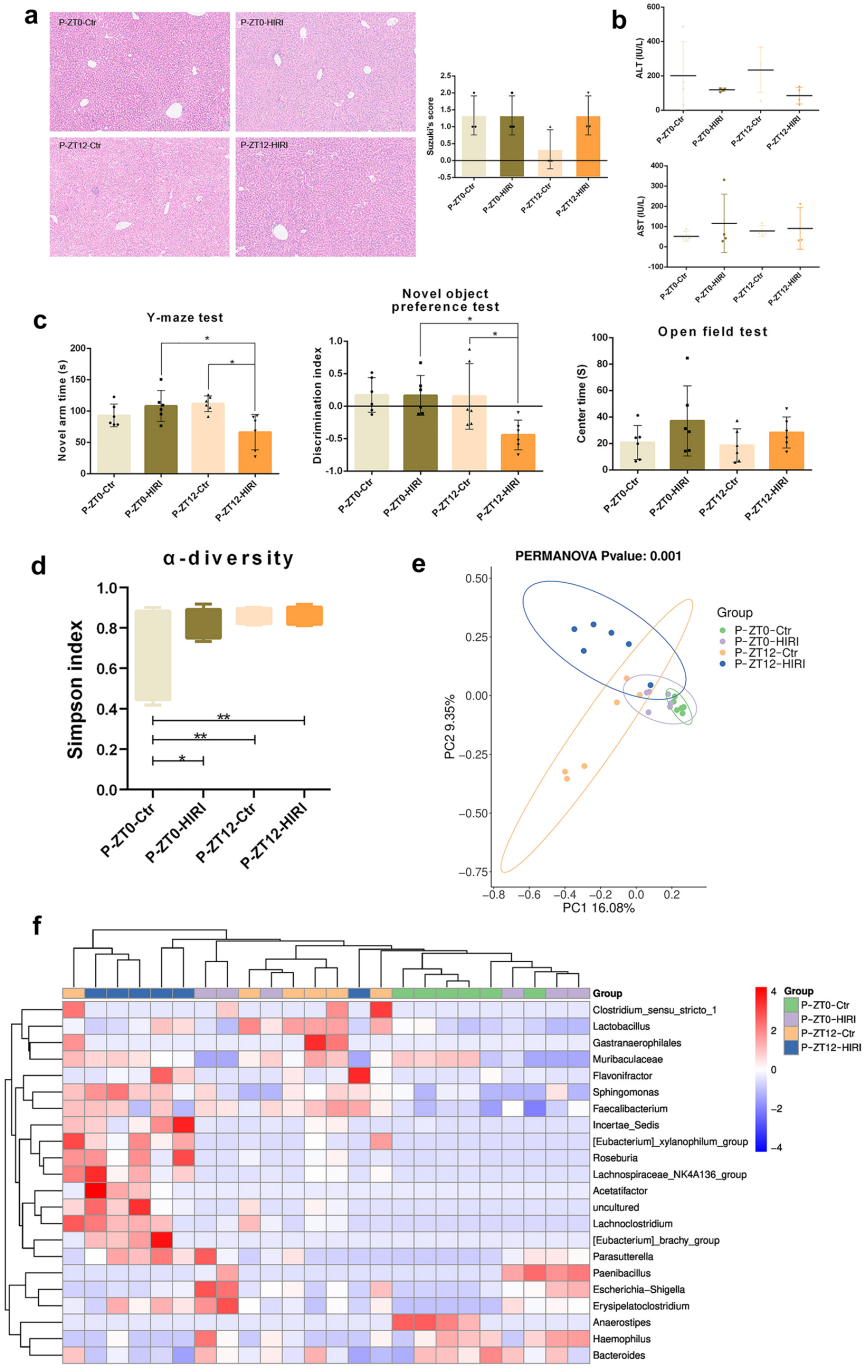
Microbiota from ZT12-HIRI mice induced cognitive impairment behavior and abnormal gut microbiota composition compared with ZT0-HIRI mice. Four groups: P-ZT0-HIRI, *n* = 6; P-ZT12-HIRI, *n* = 6; P-ZT0-Ctr, *n* = 6; P-ZT12-Ctr, *n* = 6. **A** The light microscopy and Suzuki’s score of liver tissues (n = 3). Hematoxylin and eosin (H&E). Magnification × 100. **B** Serum levels of ALT and AST (*n* = 3–4). **C** Cognitive behavior test (open-field test, Y-maze test and novel object preference test; *n* = 6). **D** Alpha Diversity (Simpson index). **E** Beta Diversity (PCoA). (**F**) Heatmap of differential species at the genus level. One-Way ANOVA (Tukey’s multiple comparisons test); *p < 0.05, **p < 0.01

To investigate whether diurnal differences in HIRI-related cognitive function are linked to gut microbiota, we established pseudo-germ-free mice and transplanted in them fecal microbiota from HIRI mice. There was no significant difference in the liver pathological score and serum ALT/AST between the four groups after FMT, but the mice receiving ZT12-HIRI FMT showed cognitive impairment compared with the P-ZT0-HIRI and P-ZT12-Ctr groups (Fig. 4A–C). For Alpha diversity, the Simpson index did not differ between the P-ZT12-HIRI and P-ZT12-Ctr or P-ZT12-HIRI and P-ZT0-HIRI (Fig. 4D). For Beta diversity, the principal component analysis (PCA) depicted significant compositional discriminations inside the gut microbiota between the P-ZT12-HIRI and P-ZT12-Ctr or P-ZT12-HIRI and P-ZT0-HIRI (Fig. 4E). Twenty two differentially expressed species were found, such as *Lactobacillus*, *Anaerostipes*, *Escherichia*–*Shigella*, *Haemophilus*, and *Sphingomonas* (Fig. 4F).

### Differences in lipid metabolomic profiles in the hippocampus of pseudo germ-free mice receiving FMT

**Fig. 5:**
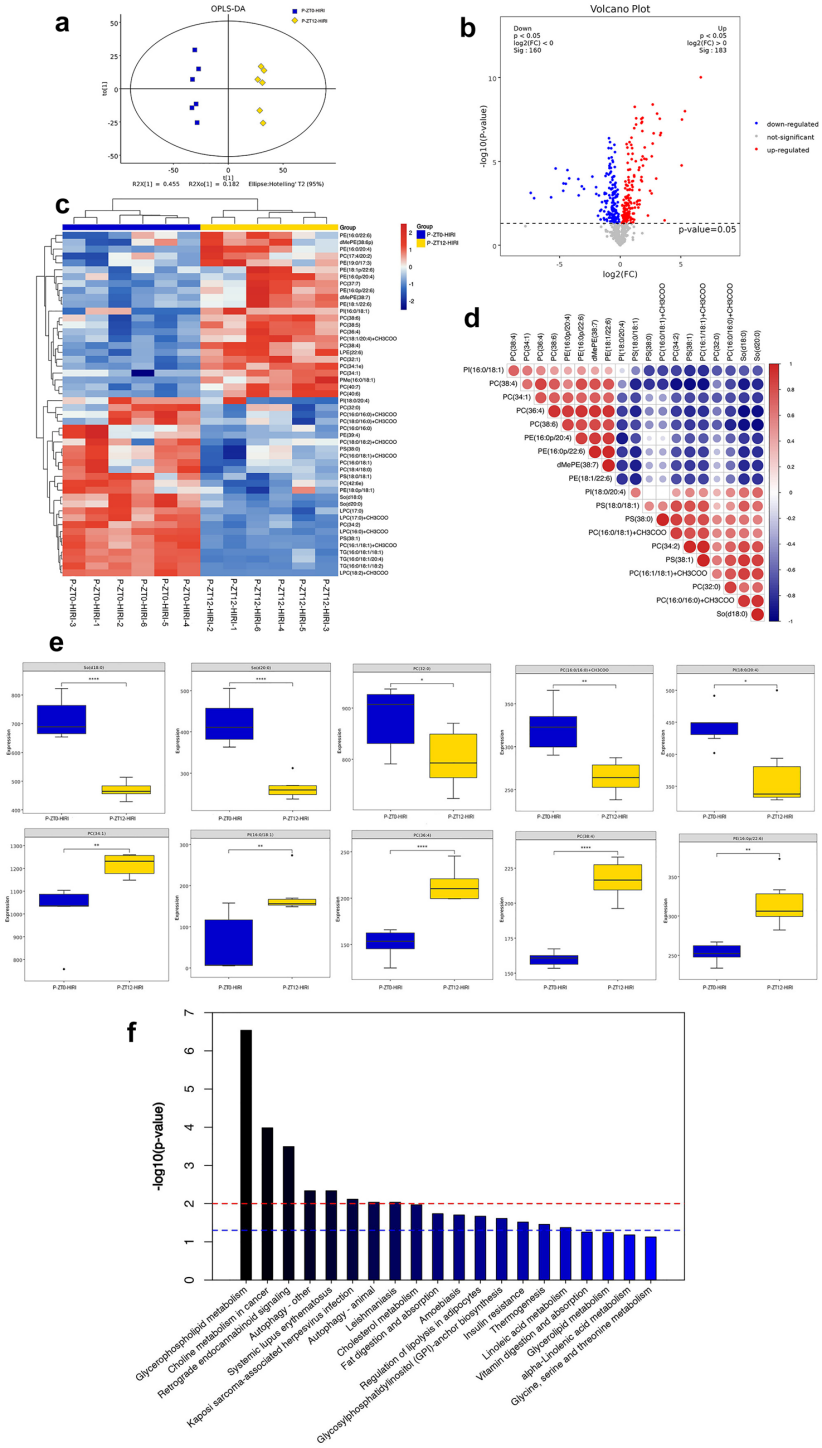
Lipidomics perturbations in the hippocampus of mice transplanted with fecal microbiota of the HIRI model. **A** PCA (principal component analysis). **B** Volcano plot of differential metabolites. **C** Cluster plot of the top 50 differential metabolites. **D** Pearson correlation coefficient of the top 20 differential metabolites. Red/Blue indicates a positive/negative correlation, respectively, and blue indicates a correlation. The size of the dots represents the magnitude of the correlation coefficient. **E** Box plot of the top different metabolites. **F** KEGG pathway enrichment analysis of differential metabolites. *PE* Phosphatidylethanolamine, *PC* Phosphatidylcholine, *PI* Phosphatidylinositol, *LPE* Lysophosphatidylethanolamine; *PMe* Phosphatidylmethanol, *PS* Phosphatidylserine, *LPC* Lysophosphatidylcholine, *TG* Triglyceride. T test; *p < 0.05, **p < 0.01, ***p < 0.001

Lipid metabolism is closely related to cognitive function, and gut microbiota can affect lipid metabolism through a variety of ways [14, 15]. In addition, the hippocampus is involved in cognitive modulation, anatomically. Meanwhile, we found that there was no significant difference in hippocampal levels of inflammatory cytokines (IL-1β, TNF-α, and NF-κB) between different groups (Supplementary Fig. 1). Given this, LC–MS was used to analyze the differences in hippocampal lipid metabolism between the P-ZT0-HIRI and P-ZT12-HIRI groups. There were significant differences in OPLS-DA scores between the two groups (Fig. 5A). Compared with the P-ZT0-HIRI, a total of 78 lipid molecules with significant differences were detected in the ZT12-HIRI, including 34 which were up-regulated and 44 down-regulated (Fig. 5B). We ranked the differential metabolites based on the VIP and performed a cluster analysis of the top 50 (Fig. 5C) and a correlation analysis of the top 50 (Fig. 5D). The top 5 up-regulated metabolites of the P-ZT12-HIRI group according to the VIP were PC(34:1), PI(16:0/18:1), PC(36:4), PC(38:4), and PE(16:0p/22:6), while the top 5 down-regulated metabolites were So(d18:0), So(d20:0), PC(32:0), PC(16:0/16:0) + CH3COO, and PI(18:0/20:4), respectively (Fig. 5E). In addition, the KEGG pathway enrichment analysis was performed on differential metabolites, which were found to be significantly enriched in lipid metabolism pathways, such as glycero-phospholipid metabolism, cholesterol metabolism, fat digestion and absorption, and regulation of lipolysis in adipocytes (Fig. 5F).

## Discussion

Liver is a major metabolic organ as well as the main peripheral circadian organ, and its damage may lead to dyshomeostasis of extrahepatic organs, including the central nervous system and gut [16, 17]. Our current study showed that HIRI caused significant liver pathological damage and induced cognitive impairment, which is consistent with earlier studies [18]. Interestingly, we illustrated that cognitive impairment caused by HIRI underwent diurnal oscillations. Specifically, the composition of gut microbiota had significant difference between the HIRI and control groups as well as diurnal differences between the ZT0 and ZT12 groups. Furthermore, gut microbiota transplantation from the ZT12-HIRI was shown to transmit cognitive impairment behavior. These findings suggest a direct association between gut microbiota and the diurnal variation of HIRI-related cognitive dysfunction.

In recent years, the circadian rhythm has attracted tremendous attention due to its substantial role in regulating physiology and behavior. Evidence from animal experiments shows that HIRI is more severe in the evening when compared with the morning after 90 min of ischemia and 6 h of reperfusion [8]. Nevertheless, clinical data from Ren et al. suggest that circadian rhythms have a significant impact on surgical outcomes of liver transplantation for patients [19]. Our current data, however, indicate that the histopathologic changes and serum ALT/AST had no significant difference in the evening (ZT12-HIRI) when compared with the morning (ZT0-HIRI) after 90 min of ischemia and 72 h of reperfusion. What did differ was HIRI-related cognitive functions. Compared with the ZT0-HIRI, the ZT12-HIRI group performed poorly on the Y-maze test and the novel object preference test due to impaired spatial working and reference memory and non-spatial visual learning memory. The observed change in histopathology and serum transaminase levels between the ZT0 and ZT12 in this study is not consistent with the study conducted by Chen et al. [8]. We speculate that this might be due to different reperfusion times.

Gut microbiota, in both mice and humans, exhibit diurnal oscillations that are influenced by feeding rhythms, and involved in the regulation of a variety of pathophysiological activities [20, 21]. The compositions of gut microbiota and metabolites, such as SCFAs, secondary bile acids (Bas), and 5-hydroxytryptamine (5-HT), are closely related to systemic metabolism and function, and related studies have shown that gut microbiota have potential neuroprotective effects [22]. We showed that the composition of gut microbiota had diurnal differences between the ZT0-HIRI and ZT12-HIRI. The limitation is that our current results can only suggest that alterations in the gut microbiota may be the result of a combination of hepatic ischemia–reperfusion and circadian rhythms. To further explore the relationship between gut microbiota and diurnal differences in HIRI-related cognitive function, we established pseudo-germ-free mice and transplanted in them fecal microbiota from HIRI mice. We found that gut microbiota transplantation from the ZT12-HIRI influenced cognitive impairment behavior. Microbial metabolites are one of the important mechanisms for gut-to-brain communication [23]. We analyzed the differences in metabolites of gut microbiota between the ZT0-HIRI and ZT12-HIRI, and a total of 387 metabolites of intestinal flora with significant differences were detected in the ZT12-HIRI compared with the ZT0-HIRI, including 183 which were up-regulated and 204 down-regulated. Among them, some differentially expressed metabolites, such as LysoPE, PC, and LysoPC, had been shown to be involved in the regulation of cognitive impairment [24]. The KEGG pathway enrichment analysis demonstrated that the differential metabolites were significantly enriched in lipid metabolism pathways, such as the glycero-phospholipid metabolism, sphingolipid metabolism, alpha-linolenic acid metabolism, and fat digestion and absorption. In addition, to investigate whether the differences in gut microbiota composition and metabolites between ZT0 and ZT12 were related to feeding, we calculated the food consumption of two groups (ZT0-HIRI vs. ZT12-HIRI) during the first 72 h of reperfusion and found that there were no significant differences in food intake among them (Data are not presented in this article). However, since we do not further compare the gut microbiota and metabolites between the two groups of pseudo-germ-free that had been kept under the same primary condition following FMT, we cannot fully determine the interference of feeding on the experimental results.

Previous studies have associated liver-related cognitive impairment with blood ammonia concentration and hippocampal inflammatory factors [3]. Our experimental data showed that there were no significant differences in blood ammonia concentration and hippocampal levels of inflammatory cytokines (IL-1β, TNF-α, and NF-κB) between ZT0-HIRI and ZT12-HIRI groups as well as P-ZT0-HIRI and P-ZT12-HIRI groups. So we speculated that there may be other mechanisms affecting circadian oscillation in HIRI-related cognitive function. Lipid metabolism is closely related to liver metabolism, gut microbiota, and cognitive function [25]. Compared to conventional mice, germ-free mice on chow-diet display reduced levels of multiple lipid molecules, concomitant with increased liver cholesterol, and decreased triglyceride content [14]. Cross-validation analysis of data from a Life-Lines-DEEP study reported that microbiota explained 4.5% of the variance in body mass index, 6% in triglycerides, and 4% in high-density lipoproteins [26]. In terms of cognitive function, a study by Bowers et al. indicated that lipid metabolism disorder in the hippocampus is a key factor affecting neural stem/progenitor cell activity and cognitive function [15]. Data from Li et al. also indicated that neuronal cholesterol metabolism in the hippocampus is closely related to memory histone acetylation-mediated memory [27]. By analyzing lipid metabolism in the hippocampus of FMT treated germ-free mice, we found a large number of differentially expressed lipid molecules between the P-ZT0-HIRI and P-ZT12-HIRI. Compared with the P-ZT0-HIRI, a total of 78 lipid molecules with significant differences were detected in the ZT12-HIRI, including 34 which were up-regulated and 44 down-regulated, suggesting that gut microbiota might affect cognitive function in HIRI mice by altering lipid metabolism in the central nervous system. As a limitation, our current study does not examine hippocampal circadian genes and signaling pathways, such as the light signal and the CLOCK genes, which have previously linked circadian oscillations, and hippocampus and cognitive functions [28–30].

## Conclusions

To summarize, our findings show that HIRI-related cognitive impairment undergoes diurnal oscillations, and gut microbiota may be involved in circadian differences of cognitive function by affecting hippocampal lipid metabolism. Our current study provides new insights into the “liver-gut-brain” axis in the context of HIRI.

### Supplementary Information

Below is the link to the electronic supplementary material.

Supplementary file1 (DOCX 113 KB)

## Data Availability

All relevant data supporting the findings of this study are available from the corresponding author on reasonable request.
