# Supplementary material for: Gut microbiota regulates circadian oscillation in hepatic ischemia–reperfusion injury-induced cognitive impairment by interfering with hippocampal lipid metabolism in mice
Source: Hepatol Int. 2023 Apr 1;17(6):1645–58. doi: 10.1007/s12072-023-10509-w (PMC10661774; doi:10.1007/s12072-023-10509-w)
Supplement: Supplementary file 1 — Supplementary file1 (DOCX 113 KB) [file 12072_2023_10509_MOESM1_ESM.docx]

**Fig. 1.** Levels of hippocampal inflammatory cytokines (ELISA): **(ACE)** Hippocampal levels of IL-1β, TNF-α and NF-κB between ZT0-HIRI (n=4) and ZT12-HIRI (n=4) groups. **(BDF)** Hippocampal levels of IL-1β, TNF-α and NF-κB between P-ZT0-HIRI (n=4) and P-ZT12-HIRI (n=4) groups. IL-1β, interleukin-1β; TNF-α, tumor necrosis factor-α; NF-κB, nuclear factor κB. T test.
